# Detailed statistical analysis plan for a guided self-determination intervention versus an attention control for outpatients with type 2 diabetes in the randomised OVERCOME trial

**DOI:** 10.1186/s13063-024-08589-6

**Published:** 2024-11-11

**Authors:** Anne Sophie Mathiesen, Vibeke Zoffmann, Jane Lindschou, Janus Christian Jakobsen, Christian Gluud, Markus Harboe Olsen, Bodil Rasmussen, Emilie Haarslev Schröder Marqvorsen, Mette Juel Rothmann

**Affiliations:** 1 Department of Nephrology and Endocrinology, Center for Cancer and Organ Diseases, Copenhagen University Hospital - Rigshospitalet, Copenhagen, Denmark; 2 Steno Diabetes Center Odense, Odense University Hospital, Odense, Denmark; 3 The Interdisciplinary Research Unit of Women’s, Children’s and Families’ Health, The Julie Marie Center, Copenhagen University Hospital - Rigshospitalet, Copenhagen, Denmark; 4 Department of Public Health, Sector of Health Services Research, University of Copenhagen, Copenhagen, Denmark; 5 School of Nursing and Midwifery, Faculty of Health, Deakin University, Melbourne, Australia; 6 Centre for Clinical Intervention Research, Copenhagen Trial Unit, The Capital Region, Copenhagen University Hospital - Rigshospitalet, Copenhagen, Denmark; 7 Department of Regional Health Research, The Faculty of Heath Sciences, University of Southern Denmark, Odense, Denmark; 8 Department of Neuroanaesthesiology, The Neuroscience Centre, Copenhagen University Hospital - Rigshospitalet, Copenhagen, Denmark; 9 Department of Clinical Research, Faculty of Health Science, University of Southern Denmark, Odense, Denmark

**Keywords:** Diabetes, Guided self-determination, Self-determination theory, Autonomy-supportive, Diabetes distress, Depression, HbA1c

## Abstract

**Background:**

Autonomy-supporting interventions may be a prerequisite to achieve better long-term management of type 2 diabetes. Evidence suggests that the guided self-determination (GSD) method might improve haemoglobin A1c and diabetes distress in people with type 1 diabetes. The evidence of an effect of a GSD intervention compared with an attention control group in adults with type 2 diabetes is unknown.

**Methods/design:**

The trial is designed as a pragmatic, investigator-initiated, dual-centre, randomised, parallel-group, assessor-blinded, superiority clinical trial of persons with type 2 diabetes. A nurse will administer GSD intervention versus an attention control. The primary outcome is diabetes distress, and secondary outcomes are quality of life, depressive symptoms, and non-serious adverse events. Exploratory outcomes are haemoglobin A1c, motivation, and serious adverse events. Participants are assessed at baseline, 5-, and 12-month follow-up. Here, we present a detailed, comprehensive plan of all statistical analyses, including methods to handle missing data, and assessments of the underlying statistical assumptions. The statistical analyses will be conducted independently by two statisticians following the present plan.

**Discussion:**

To mitigate the risk of analysis bias and increase the validity of the OVEROME trial, this statistical analysis plan was developed prior to unblinding of the trial results in concordance with the Declaration of Helsinki and the Conference on Harmonization of Good Clinical Practice Guidelines.

**Trial registration:**

ClinicalTrials.gov NCT 04601311. Registered on October 2020.

**Supplementary Information:**

The online version contains supplementary material available at 10.1186/s13063-024-08589-6.

## Background

Type 2 diabetes management aims to prevent and reduce complications of diabetes. Diabetes self-management care depends among other factors on the motivation and autonomy of the person with diabetes. Accordingly, autonomy-supporting interventions might be crucial in achieving engagement and long-term improvement in people with type 2 diabetes through shared decision-making and collaborative goal setting [1]. A method designed to promote autonomy and diabetes self-management skills in people with type 2 diabetes is the empowerment-based method, the GSD method [2–4]. Evidence suggests that the GSD method might improve serum haemoglobin A1c (HbA1c) and reduce diabetes distress in people with type 1 diabetes [5]. However, the effect of GSD on diabetes distress in people with type 2 diabetes is currently unclear and existing evidence is prone to systematic as well as random errors [6, 7].

The OVERCOME trial is a pragmatic, investigator-initiated, dual-centre, randomised, parallel-group, assessor-blinded, superiority clinical trial of persons with type 2 diabetes, designed to assess the benefits and harms of GSD applied in outpatient clinics [6]. It is the recommendation of the Helsinki Declaration [8] and the International Conference on Harmonization of Good Clinical Practice (ICH-GCP) that clinical trials are analysed according to a predefined statistical plan to counteract data-driven analysis results and selective reporting. Here, we describe the detailed statistical analysis plan for the primary, secondary, and exploratory outcomes in the OVERCOME trial [6]. The main publications of the trial results will adhere to this plan.

## Methods

For a detailed description of the trial design and methods, we refer to our trial protocol [6]. The trial population are persons with type 2 diabetes treated at the Department of Endocrinology, the University Hospital of Copenhagen—Rigshospitalet, The Capital Region of Denmark and Steno Diabetes Centre Odense, Odense University Hospital, The Southern Region of Denmark. The participants are eligible for inclusion if they fulfil the inclusion and none of the exclusion criteria below.

Inclusion criteria:

- Being 18 years of age or older.
- Having been diagnosed with type 2 diabetes for 3 months or more according to the International Classification System of Diseases (ICD11.2–11.9).
- Having signed informed consent.

Exclusion criteria:

- Pregnancy: women who are premenopausal will be asked if they are pregnant or are planning pregnancy prior to inclusion.
- Prior participation in GSD course(s) for the past 2 years.
- Lack of signed informed consent.

The trial adheres to our published protocol [6] and the Helsinki Declaration. The trial has been registered at ClinicalTrials.gov (NCT04601311, October 2020) and the Danish Data Protection Agency (P-2020–864, January 2020). The Ethics Committee of the Capital Region of Denmark reviewed the trial protocol but exempted the trial protocol from full review (H-20003638, August 2020). For reporting of this statistical analysis plan, we adhered to the ‘Guidelines for the Content of Statistical Analysis Plans in Clinical Trials’ [9].

### Randomisation and blinding

Participants are randomised centrally at a 1:1 ratio using a web-based system developed and administered by the Copenhagen Trial Unit. The allocation is computer-generated through permuted blocks of varying sizes and concealed from the investigators. Due to the nature of the intervention, the allocation is not blinded to the investigators. The certified GSD nurses enrol the participants and assign the intervention. The randomisation is stratified according to centre and sex.

Participants and treatment providers will not be blinded to the allocated trial intervention due to the difficulties of blinding psychological interventions [10]. The treatment providers will not be involved in the analyses. The outcome assessors, the statisticians at the Copenhagen Trial Unit, and the decision-makers regarding the reporting and conclusions will be blinded to the participants’ allocation.

## Trial interventions

### Experimental intervention

The GSD is a theory-based problem-solving method to overcome barriers related to diabetes management and collaborative care [3]. The method strives to strengthen autonomous motivation, empowerment, and self-determination by focusing on life-illness integration, the relational potential for change, and shared decision-making [4].

The focus areas of the method are embedded in the reflection sheets for the person with type 2 diabetes to work with at home or online. Experienced nurses are certified in the GSD method including advanced communication techniques such as mirroring, active listening, and value-clarifying questions to promote autonomous reflection. Experienced diabetes nurses certified in the GSD method provide two to five sessions every second week after randomisation, as a need-based and thus stepped-care intervention to each participant individually. The sessions are conducted face to face, by video, or over the telephone. The stepped-care intervention is provided as a digital version, an analogue GSD (in paper), or a mixed version as preferred by the participant. The GSD intervention requires participants to complete 13 reflection sheets in a predefined order as preparation for five individual sessions [6]. For further information, we refer to our protocol [6].

### Control intervention

The participants in the control group receive an attention control intervention that include two to five needs-based sessions with a communication-trained medical student or a psychology student, who were all certified counsellors. The needs-defined sessions are scheduled concurrently with the sessions in the experimental group, namely 2 weeks, 4 weeks, and 6 weeks after randomisation. All sessions last up to 1 h. The sessions are conducted face to face, by video, or over the telephone [6].

### Baseline data

Socio-demographic data (age, sex, civil status, educational level [11], employment status, diabetes-related comorbidities, psychiatric comorbidities, medical comorbidities, and diabetes medication) will be presented in a table.

### Outcomes

According to our protocol, outcomes were defined as primary, secondary, or exploratory. This paper describes the detailed analysis plan for all outcomes.

Primary outcome:

- Diabetes distress assessed by the validated 20-item scale of diabetes-related distress burden, problem areas in diabetes (PAID) [12], at 12 months after randomisation.

Secondary outcomes:

- Depressive symptoms assessed by the hospital anxiety and depression scale (HADS) [13], at 12 months after randomisation.
- Quality of life (QOL), assessed as the SF-36 mental component score and physical component score [14, 15] at 12 months after randomisation.
- Adverse events assessed by the negative effect questionnaire (NEQ)-20 [16, 17]. NEQ-20 is assessed at 12 months after randomisation.

Exploratory outcomes:

- HbA1c concentration at 12 months after randomisation.
- Type of motivation regarding diabetes self-care practices assessed by the treatment self-regulation questionnaire (TSRQ), at 12 months after randomisation:
  - ◦ Autonomous
  - ◦ Controlled (external)
  - ◦ Resigned (amotivated)
- Proportion of participants with at least one serious adverse event in the intervention period, defined according to the International Harmonisation of Good Clinical Practice (ICH-GCP), assessed 12 months after randomisation.

### Sample size calculation

This randomised clinical trial is designed to evaluate superiority when comparing the intervention group and the attention control group. The sample size calculation was based on our primary outcome and an estimated minimal clinical important difference from the mean of six points and standard deviation (SD) of 16 points from our systematic review conducted prior to initiating this OVERCOME trial [7, 18].

With a power of 80% (a beta at 20%) and an alpha at 5%, two-tailed, a sample size of 112 participants is needed in each intervention group corresponding to a total of 224 participants included in the trial.

### Power calculations for the secondary outcomes

For the secondary outcome depressive symptoms, measured by HADS, we chose an estimated minimal important difference of three points and a SD of six points based on the total scale applied in previous trials [19, 20], which correspond to a power of 96.3%.

For the secondary outcome QOL, measured by SF-36, we chose an estimated minimal important difference of six points and SD of 15 points based on previous trials [14, 15], which correspond to a power of 84.9%.

For the secondary outcome adverse events, measured by NEQ-20, we pragmatically chose an estimated minimal important difference of five points and a SD of 13 points [16, 17], which correspond to a power of 82.1%.

### General analysis principles

Statistical analyses will be performed using the latest stable version of R (R Core Team, Vienna, Austria) and/or Stata (StataCorp, LLC, TX, USA) [21]. All valid assessments from every randomised participant will be analysed according to the intention-to-treat principle. Discontinuations and drop-outs and the reasons for these will be reported. Though hypothesis-generating only, we will consider performing a per protocol analysis, if the number of participants who drop-out prematurely exceeds 5% of the total trial population. In the per protocol analysis, which is exploratory, we will include all participants that attended ≥ 2 visits according to our definition of adherence (see section ‘ Handling of missing data’).

### Statistical analysis

#### Continuous outcomes

Continuous data will be presented as means and 95% confidence intervals in figures and tables. The outcomes will be analysed using linear regression analyses adjusted for baseline values of the outcomes: diabetes distress, depressive symptoms, quality of life, HbA1c, motivation (autonomous, controlled, resigned), and the stratification variables (centre and sex).

#### Dichotomous outcomes

Dichotomous outcomes will be presented as proportions for each group and will be analysed assuming a binomial distribution using the generalised linear model with a log link adjusted for centre and sex. If the analysis does not converge, binary data will be analysed using logistic regression, and the NLCOM command [21] will be used to obtain relative risks (RRs) and 95% confidence intervals of the RRs. Similar methods will be used for analysis in R; however, 95% confidence intervals will be bootstrapped.

#### Handling of missing data

Missing data will be handled according to the recommendations of Jakobsen et al. [22]. In short, all randomised participants (the intention-to-treat population) will be included in the primary analysis of all outcomes. If the missing data exceeds 5%, we will consider using multiple imputations and perform the best–worst/worst-best case scenarios to assess the potential range of impact of the missing data. As this is a stepped care intervention, adherence to the interventions is defined as having attended two–five sessions. Any deviations from protocol will be summarised in the section ‘deviations between the protocol and the trial’.

#### Early stopping

If we are not able to recruit and randomise the full sample size of 224 participants, the statistical significance level will be adjusted according to the Lan-DeMets sequential monitoring boundaries based on O’Brien-Fleming alpha-spending function [23–26]. The Lan-DeMets-O’Brian-Fleming method for sequential monitoring is able to take the accrued sample size distance to the planned sample size into consideration to reduce the risks of false positive findings if we are not able to achieve the planned sample size [26, 27]. We will only use this analysis once if we do not reach our sample size.

This will be applied for the primary and secondary outcomes: diabetes distress, depressive symptoms, and quality of life. Similarly, the primary confidence intervals presented will be the adjusted confidence interval. Furthermore, we will use the minimal important differences defined above. Finally, outcomes where adjusted confidence intervals are presented will also have 95% confidence intervals presented in the supplemental material.

#### Correction for multiplicity

We assess only one primary outcome and consider all other outcome results as hypothesis-generating only. Therefore, we have used a two-sided alpha of 5% as the acceptable risk of type I error in the sample size and power estimations.

#### Assessment of underlying statistical assumptions

We will assess the statistical assumptions underlying each statistical method following the commendations of Nørskov et al. [28]. In short, for all regression analyses, we will test for major interactions between each covariate and the intervention variable. When assessing for major interactions, we will, in turn, include each possible first-order interaction between included covariates and the intervention variable. For each combination, a significant interaction is only evident if the interaction is statistically significant after Bonferroni adjusted thresholds (0.05 divided by the number of possible interactions, if any interaction exits).

For the assessment of the underlying statistical assumptions for the linear regression, we will inspect quantile–quantile plots of the residuals for normal distribution and assess for homogeneity of variances by using residuals plotted against covariates and fitted values. If deviations from model assumptions are present, we will consider log or square root transformation.

To consider a potential overdispersion, we will assess if the deviance divided by the degrees of freedom is significantly > 1.0. In this case, we will consider using a maximum likelihood estimate of the diversion parameter.

#### Subgroup analyses

We will conduct the following subgroups analyses on our primary outcome diabetes distress: sex (men compared to women), site (Rigshospitalet compared to Steno Odense), educational level (bachelor’s degree or master’s degree compared to < bachelor’s degrees), comorbidities (participants with ≤ 2 comorbidities compared to participants with ≥ 3 comorbidities), and duration of diabetes (< 10 years compared with ≥ 10 years). The subgroup analyses will be illustrated in forest plots.

#### Statistical reports

Two independent statisticians at the Copenhagen Trial Unit will analyse blinded data on all outcomes with the intervention groups concealed as ‘A’ and ‘B’. Two independent statistical reports will be handed over to the principal investigator as well as shared with the steering group. If any discrepancies between the two primary statistical reports, these will be identified, and the steering committee will consider which is the most correct result and arguments for the choice will be added to the final publication. A final statistical report will be prepared, and the two (or potentially three, if anything is to be corrected) reports will be published as supplemental material.

## Discussion

In this paper, we described a predefined statistical analysis plan for the OVERCOME trial, which will be published before unblinding of the trial data. The statistical analysis plan will limit selective reporting, data-driven interpretation, and thus increase the validity of our results.

Large parts of the Danish society were shut down due to COVID-19 from March 20, 2020, and onwards during the OVERCOME trial intervention and follow-up period. The shutdowns from COVID-19 may potentially have impacted the recruitment rates, discontinuations, and drop-out rates [29].

During COVID-19, a substantial additional workload was imposed on the clinical personnel, including nurses in the outpatient clinics where the OVERCOME trial was hosted. Several nurses involved in the OVERCOME trial were moved to COVID-19 departments to care for hospitalised persons with the virus. This may have inferred disruption in some of the GSD trajectories.

Following the shutdown due to COVID-19, all nurses in Denmark had a national strike lasting 2 months, which may have delayed the recruitment of participants and might have compromised the quality of deliverance of the GSD intervention [30].

### Strengths

The OVERCOME trial was designed to minimise systematic as well as random errors. We conducted a systematic review [7] prior to initiating the trial, which enabled us to derive anticipated intervention effects for calculating the sample size and the estimated minimal clinical important differences for power calculations for the secondary outcomes. We reduced the risk of selective outcome reporting by predefining all outcomes in our trial protocol [6]. The statistical analysis plan ensures methodological transparency, enables reproducibility, and will minimise selective reporting bias. Publishing statistical analyses plans prior to publishing the results of the trial are reported to be necessary [31]. We applied an attention control group which reduces the risk that a potential effect of the intervention may be due to the Hawthorne effect [32].

### Limitations

The primary limitation is the high risk of missing data in clinical trials including persons with multimorbidity and psychiatric disorders [33]. We have tried to explicitly address this by describing our predefined plan for multiple imputation which we will most likely be compelled to conduct. Another limitation is the superiority design in which firm conclusions can only be derived if statistical differences between groups are found on the primary outcome. But even without statistically significant differences, the trial may still contribute to accumulating evidence across trials in, e.g. systematic reviews and meta-analyses. The motivation for treatment and the therapeutic alliance might influence the efficiency of the interventions but we did not assess these variables. A baseline assessment of these variables would have allowed us to assess a potential effect modification on the interaction between the two variables and the effect of the intervention. At the design stage of this trial, we tried to account for the fact that it is not possible to blind participants and providers in psychological interventions. We argue that we considered the limitations regarding blinding of all psychological trials thoroughly (see, e.g. Juul et al.) [10], but acknowledge that any differences between groups may be due to bias, even in our set up trying to reduce such risks.

We attempted to blind external statisticians by concealing groups as ‘A’ and ‘B’ but are aware that this procedure does not blind statisticians fully.

## Trial status

The recruitment initiated in November 2020 and has been finalised. The collection of follow-up data will be completed in January 2024.

## Conclusion

In line with the Declaration of Helsinki and the International Conference on Harmonization of Good Clinical Practice Guidelines, we have developed this statistical analysis plan with the aim of decreasing the risk of selective reporting and analysis bias and thereby increase the validity of the OVERCOME trial.

## Supplementary Information

Supplementary Material 1

## Data Availability

The datasets analysed from the OVERCOME trial are planned to be made available at Zenodo.org, when data collection is finalised.

## Abbreviations

GSD: Guided self-determination method
HADS: Hospital anxiety and depression scale
HbA1c: Serum haemoglobin A1c
NEQ: Negative effects questionnaire
PAID: Problem areas in diabetes
QOL: Quality of life
TSRQ: Treatment self-regulation questionnaire
